# Elucidating gut microbiota–hippocampus interactions in emerging psychosis: A new perspective for the development of early interventions for memory impairments

**DOI:** 10.3389/fpsyt.2023.1098019

**Published:** 2023-03-23

**Authors:** Galya C. Iseli, Sarah Ulrich, André Schmidt

**Affiliations:** Department of Clinical Research (DKF), University Psychiatric Clinics (UPK), Translational Neurosciences, University of Basel, Basel, Switzerland

**Keywords:** psychosis, gut microbiota, hippocampus, brain-gut interactions, memory, early detection and interventions, schizophrenia

## Abstract

Hippocampal dysregulation might be a key pathophysiological factor for memory impairments in psychosis. Contemporary models particularly postulate that an imbalance of hippocampal glutamate and GABA leads to impaired memory and may thus serve as a therapeutic target to improve memory deficits. However, currently available interventions in early stages of psychosis do not explicitly target hippocampal pathology. A novel approach for manipulating hippocampus-dependent memory processes is provided *via* the gut microbiota. In this perspective article, we first recapitulate compelling evidence for emerging hippocampus pathology during the development of psychosis. The following sections emphasize the critical role of the gut microbiota in hippocampus plasticity and memory, and summarize existing evidence of gut microbiota alterations in different stages of psychosis. Finally, we propose a novel conceptual roadmap for future studies deciphering gut microbiota–hippocampus synergisms in emerging psychosis and argue that specific microbial supplementation might be promising for improving hippocampus-dependent memory deficits in early stages of psychosis.

## Introduction

The hippocampus is crucial for episodic memory (1) and deficits in this function have been associated with subsequent transition to frank psychosis (2) and functional disability (3) in clinical high-risk (CHR) individuals. Reduced gray matter volume in the hippocampus is one of the most robust neuroimaging findings in patients with schizophrenia (4), a finding that is also evident in their non-psychotic relatives (5), patients with first-episode psychosis (FEP) (6, 7), and CHR subjects (8, 9), especially in those with the later transition to psychosis (10). It has further been shown that smaller hippocampus volume is related to poorer verbal memory performance in schizophrenia patients (11) and predictive of transition to schizophrenia at a 2-year follow-up in patients with early psychosis (12). Patients with schizophrenia (13), early psychosis (14), FEP (15), and those at CHR (16) do also exhibit reduced hippocampus activation during episodic memory processing. Reductions in hippocampal activation and volume have been associated with impaired global cognition, symptom severity (17, 18) and predicted by negative and disorganized schizotypal symptoms in non-clinical samples (19). This suggests hippocampus pathology to be prevalent early during the psychosis continuum, thus potentially serving as a predictive marker for disease progression and target for novel approaches to early intervention.

## Hippocampus pathology in psychosis–underlying mechanisms

A compelling line of evidence for hippocampal dysfunction comes from a neurodevelopmental model of schizophrenia, the methylazoxymethanol acetate (MAM) model (20). A key abnormality in the MAM model is a deficit expressed in the parvalbumin-expressing gamma-aminobutyric acid (GABA)ergic inhibitory interneurons of the hippocampal region in mice. This augmented hippocampal function drives the elevated striatal dopamine associated with psychosis *via* a polysynaptic pathway involving altered GABAergic and glutamatergic neurotransmission within the hippocampus-striatum-midbrain network (20–22). Intriguingly, administration of a positive allosteric modulator of the a5GABA_*A*_ receptor and a group 2 metabotropic glutamate receptor (mGluR2/3) agonist normalized hippocampal hyperactivity and striatal dopamine dysfunction after having developed in the MAM model (23, 24).

In support of the model, hyperperfusion of the hippocampus was reported in patients with full psychosis (25, 26) and CHR individuals (27, 28). Notably, CHR subjects who experienced remission showed a longitudinal reduction in hippocampal perfusion (28) and those who later converted to frank psychosis showed hippocampal hyperperfusion which was predictive of hippocampal volume loss (25). Increased resting perfusion of the hippocampus is already evident in non-clinical samples with high schizotypy (29) and related to delusional thinking and distress in non-help seeking individuals from the general population (30). A recent study in patients with early psychosis further showed that increased hippocampal blood flow was inversely related to task-related activation during scene processing in the anterior hippocampus (31). This finding suggests that baseline hippocampal hyperactivity in early psychosis patients appears to limit effective recruitment of this region during task performance.

In accordance with the MAM model, elevated hippocampal glutamate levels have been observed in unmedicated patients with schizophrenia (32, 33), first-episode schizophrenia (34), FEP patients with >12 months duration of untreated psychosis (6) and CHR individuals (35, 36). One study in CHR subjects also reported an inverse relationship between visuospatial ability and hippocampal glutamine concentrations (36) and another showed a trend of negative relationships between hippocampus activation during episodic memory and hippocampal glutamate concentrations (37). Moreover, CHR individuals who developed psychosis also show higher hippocampal glutamate levels compared with those who did not become psychotic (38). Hippocampus abnormalities along the progression of psychosis are summarized in Supplementary Table 1.

In summary, episodic memory deficits are evident early in the psychosis continuum and are predictive of adverse clinical outcomes in high-risk individuals. Those deficits correspond with insufficient task-related hippocampus activation, caused by hippocampal hyperactivity that results from an imbalance of excitation/inhibition (glutamate/GABA) which contributes to subsequent hippocampal volume loss (39, 40).

## Gut microbiota–hippocampus synergisms

### Regulation of neurogenesis, plasticity, and memory performance

**FIGURE 1:**
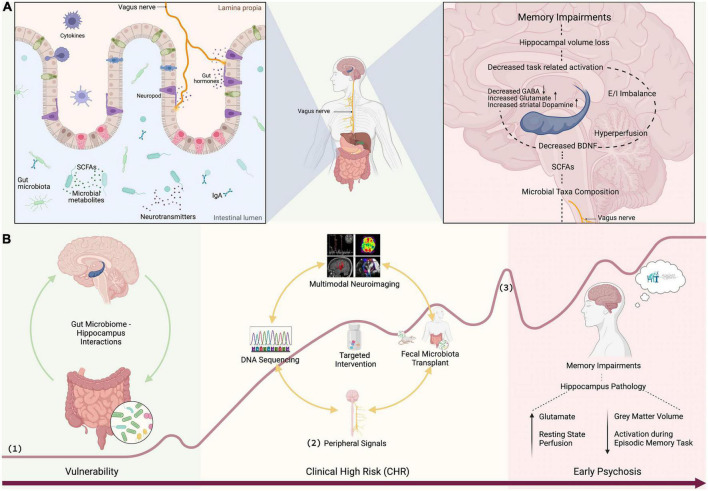
**(A)** SCFAs produced by bacterial commensals in the gut signal the brain *via* vagus nerve or neurotransmission influencing glutamate and GABA synthesis (54). Vagal sensory signaling enhances memory (49), facilitates neurogenesis, and increases the expression of BDNF (50, 51). A decrease in the BDNF, which regulates hippocampal neuroplasticity (46), decreases learning and memory functioning (47). GABAergic and glutamatergic neurotransmission drives elevated striatal dopamine (20–22). Hippocampal hyperperfusion predicts hippocampal volume loss (26). Hippocampal hyperactivity results in excitation/inhibition (glutamate/GABA) imbalance which contributes to hippocampal volume loss (39, 40) leading to memory impairment. **(B)** Discovery of gut microbiome markers: (1) curve of psychosis progression adapted from Fusar-Poli et al. (118) and Schultze-Lutter et al. (119), (2) peripheral signals such as stress (e.g., cortisol, sirtuins), inflammation (e.g., CRP, IL, TNF-α), plasticity (BDNF), and vagal signaling (e.g., ghrelin), and (3) first psychotic episode. Figure 1 was created with BioRender.com.

The presence of microbes is crucial for the development of hippocampus-dependent memory (41) and even adult hippocampal neurogenesis can be regulated by the gut microbiota (42). Recent studies in laboratory animals have shown that gut microbes drive individual differences in memory (43) and that diet-induced increases in microbial diversity improved performance on tests of working and spatial memory (44). Adult mice treated with antibiotics showed decreased hippocampal neurogenesis and memory retention that could be reversed with probiotics (45). A decrease in the Brain-derived neurotrophic factor (BDNF), a neurotrophin which regulates hippocampal neuroplasticity (46), and thereby learning and memory functioning (47), was found in the hippocampus of germ-free mice (48). Furthermore, gut vagal sensory signaling enhances memory (49), facilitates hippocampal neurogenesis and increases hippocampal expression of BDNF (50, 51), as well as regulates hippocampus function through multi-order pathways (52). Although the specific mechanisms are largely unknown, short-chain fatty acids (SCFA), particularly butyrates, are speculated to have a key role in gut-brain crosstalks [for an extensive review see Dalile et al. (53)]. SCFA produced by the bacterial commensals in the gut are able of signaling the brain indirectly *via* vagus nerve activation or directly *via* neurotransmission by influencing dopamine, glutamate and GABA synthesis (54) (Figure 1).

Butyrates produced in the gut can also directly be transported across the blood-brain-barrier (55). Administration of probiotics can increase butyrate-producing bacteria in the gut and the supplementation of prebiotics promotes their production of butyrates (56, 57) and possibly hippocampal plasticity through enhanced BDNF expression (56). As an antioxidant response, probiotics also have the ability to down-regulate the production of inflammatory cytokines within the hippocampus (58). It could be shown that 4-week probiotic supplementation improved hippocampal memory performance and enhanced prefrontal brain signal in healthy volunteers (59). A 12-week probiotic supplementation also improved cognitive function in older adults accompanied by increased levels of BDNF and changes in the gut microbiota (60). The most intriguing support for gut microbiota-hippocampus interactions comes from a recent fecal microbiota (FMT) transplantation study in mice (61). In this study, FMT from young to aged mice reversed brain immunity, hippocampal metabolome and transcriptome, as well as attenuated memory impairments.

To conclude, accumulating evidence from animal research and (intervention) studies in healthy volunteers reveal the critical role of the gut microbiota in the regulation of hippocampal plasticity, inflammation and hippocampus-dependent memory (62).

### Gut microbiota and mechanisms of psychosis pathology

Many risk factors for psychosis such as maternal infection, stress and malnutrition, early life stress, smoking and cannabis use, as well as urban environment and migration are associated with changes in gut microbiota [for a review see Kelly et al. (63)]. As stated above the gut microbiota also has an impact on different psychosis-relevant neurotransmitter systems within the hippocampus-striatum-midbrain network such as dopamine, GABA or glutamate (63). For instance, Heijtz et al. (64) showed that mice developmentally lacking any gut microbiota exhibit elevations in dopamine turnover in the striatum coupled with a hyperactivity phenotype. This is of particular relevance, as elevated striatal dopamine levels are evident in psychotic patients (65) and CHR individuals, especially those who later developed full-blown psychosis (66, 67). A groundbreaking study has further shown that optogenetic stimulation of gut vagal afferents mediated motivation for reward and striatal dopamine activity in mice (68), establishing a neural circuit for gut-induced reward. This resonates with previous evidence demonstrating that alterations to the gut microbiome can affect behavioral responses to psychostimulant drugs, possibly *via* altered dopamine and glutamate-related transcripts in the ventral striatum (69). Of note, decreased ventral striatum activation during reward processing has consistently been reported in patients with psychosis (70). The gut microbiome also influences GABA. Indicatively, GABA levels are decreased in the stool and blood of germ-free mice (71). Germ-free mice receiving FMT from schizophrenia patients had lower glutamate and higher GABA in the hippocampus and displayed schizophrenia-relevant behaviors (72).

Several gut bacteria are known to produce GABA, while *Lactobacillus rhamnosus* is the most cited (73, 74). Previous preclinical studies showed that *L. rhamnosus*, a butyrate-producing bacterial strain (75), increased GABA receptor levels in the hippocampus (76). Notably, the increase in hippocampal GABA receptor levels was not found in vagotomized mice, identifying the vagus as a major regulator between the gut microbiome and the brain (77). A previous magnetic resonance spectroscopy study further revealed that 4-weeks of supplementation with *L. rhamnosus* increased brain GABA and glutamate in mice (77). These findings correspond with another animal study indicating that prebiotics like fructo-oligosaccaride and galacto-oligosaccharide can increase GABA receptor gene expression in the hippocampus (78). This raises the question whether the postulated glutamate/GABA imbalance in psychosis (39, 40) is accompanied or strongly driven by gut microbiome alterations and whether they can be normalized with microbial interventions. The ability of certain gut bacteria such as *Lactobacillus brevis* DPC6108 to convert glutamate to GABA (79) is of particular interest.

### Gut microbiota composition in schizophrenia

At present, only a few studies used next-generation sequencing to explore microbiota alterations in patients with schizophrenia and even though there are inconsistent findings in alpha diversity measures so far, differences in beta diversity appear to be consistent in schizophrenia (80, 81). Furthermore, there are significant differences in microbiota composition in schizophrenia compared to healthy volunteers, as listed in Table 1 (71, 82–89). A recent meta-analysis suggests that schizophrenia and other psychiatric disorders, are associated with a reduction of anti-inflammatory butyrate-producing bacteria, while pro-inflammatory genera are enriched (81).

**TABLE 1:** Significant changes in relative abundance of microbial taxa in schizophrenia.

Level	Increased	Decreased	Not consistent
Phylum	Actinobacteria (87, 112)	Firmicutes (86, 112)	
	Proteobacteria* (84, 86, 89)		
Family	Actinomycetaceae (89, 113)	Alcaligenaceae (87, 92)	Enterobacteriaceae (72, 84, 86)
	Prevotellaceae (72, 86)	Pasteurellaceae (84)	Succinivibrionaceae (84, 86)
	Desulfovibrionaceae (89, 113)	**Lachnospiraceae** (72, 86, 89)	Enterococcaceae (84, 87)
	**Lactobacillaceae** (84, 86, 92)	Turicibacteraceae (84)	Ruminococcaceae (113, 114)
	Christensenellaceae (84)	/	Veillonellaceae (72, 84, 86, 92)
Phylum firmicutes	***Anaerotruncus*** (89, 113, 115)	*Coprococcus* (86, 89)	*Blautia* (86, 89)
	*Ruminiclostridium* (89, 113)	*Faecalibacterium* (89, 112)	*Clostridium* (84, 86, 116)
	*Coprobacillus* (84, 115)	/	*Turicibacter* (84, 113)
	*Veillonella* (84, 115)	/	*Megasphaera* (84, 86, 87)
	***Lactobacillus*** (84, 86, 92, 112, 115)	/	*Enterococcus* (84, 87, 115)
	*Acidaminococcus* (86, 115)	/	*Streptococcus* (84, 86)
	*Flavonifractor* (89, 113)	/	/
All other phyla	*Actinomyces* (89, 113)	/	*Bifidobacterium* (115, 117)
	*Collinsella* (86, 112)	/	*Bacteroides* (84, 117)
	*Eggerthella* (87, 89)	/	*Escherichia-Shigella* (84, 117)
	*Prevotella* (86, 89)		
	*Bilophila* (89, 113)		
	*Citrobacter* (86, 115)		
	*Succinivibrio* (86, 112)		
	*Methanobrevibacter* (86, 115)		

Differences were reported at least by 2 studies based on the review and meta-analysis of Nikolova et al. (81). Bold microbial taxa show evidence by more than 2 studies. * Presence of counterevidence by one study (116).

Further evidence for a relationship between gut microbial composition and schizophrenia comes from interventional studies with probiotics. In an open-label single-arm study, Okubo et al. (90) found that 4 weeks of administration of *Bifidobacterium breve* improved symptoms of anxiety and depression. Furthermore, combined probiotics and vitamin D supplementation for 12 weeks significantly improved psychotic symptoms in schizophrenia patients accompanied by decreases of C-reactive protein concentrations (91).

### Gut microbiota composition in early psychosis

Evidence of microbial alterations in early stages of psychosis comes from two studies and is thus very sparse. In a study with FEP patients, Schwarz et al. (92) found differences at the family level; Lactobacillaceae, Halothiobacillaceae, Brucellaceae, and Micrococcineae were found to be increased, whereas Veillonellaceae were decreased in FEP patients compared to controls. In subjects with FEP they found statistically significant increases among genera in, *Lactobacillus*, *Tropheryma*, *Halothiobacillus*, *Saccharophagus*, *Ochrobactrum*, *Deferribacter*, and *Halorubrum*. In contrast, *Anabaena*, *Nitrosospira*, and *Gallionella* showed decreased levels. Of note, the level of *Lactobacillus* correlated positively with the severity of positive symptoms and negatively with the degree of functioning. Furthermore, patients with the strongest microbiota changes at baseline showed poorer response after up to 12 months of antipsychotic treatment (92).

Preliminary evidence from a single study including 19 unmedicated ultra high-risk subjects and 81 high-risk individuals showed that the orders Clostridiales, Lactobacillales, and Bacteroidales and genera *Lactobacillus* and *Prevotella* were increased in ultra high-risk compared to high-risk and healthy controls (93). At the species level, only *Lactobacillus ruminis* was identified by both methods together as a significant feature in the ultra high-risk group.

Findings of the order Lactobacillales and the family Lactobacillaceae in phylum firmicutes, as well as order *Prevotella* in FEP and ultra high-risk subjects are consistent with the findings in schizophrenia (Table 1). Intriguingly, they further found an elevation in the SCFA related pyruvate synthesis in ultra high-risk subjects (93). The latter result is not surprising given that Clostridiales, *Prevotella*, and *Lactobacillus* are SCFA-producing bacteria (53). It is important to emphasize that type and concentration of SCFAs differently influence microglial activation, a marker of neuroinflammation (94). While butyrate can inhibit microglial activation (95), propionic acid/propionate (biosynthetic product of pyruvate) can promote microglial activation (96).

## Discussion and future directions

To date psychosis is associated with structural, functional and chemical alterations in the hippocampus. Compelling preclinical evidence further demonstrates strong interactions between the hippocampus and gut microbiota, suggesting that the hippocampus anomalies seen in psychosis might be related to gut microbiota alterations. In this final section, we propose potential new avenues for exploring gut microbiota–hippocampus synergisms in emerging psychosis. An overview of potential mechanistic pathways linking gut microbiota and hippocampal memory is provided in Figure 1.

### Discovery of gut microbiome markers in early stages of psychosis

The field of psychosis research has recognized the immense importance of the gut microbiome and the first seminal works have indicated alterations in the gut microbiota of patients. However, while there is evidence to suggest that the gut microbiome can influence adult hippocampal neurogenesis and impact disease progression, our current understanding of underlying mechanisms is still limited (97). Thus, more large-scale studies of the gut microbiome using next-generation state-of-the-art sequencing in human subjects throughout the psychosis continuum are required (Figure 1). Of particular interest are SCFA-producing bacteria, which are known to mediate cognitive processes (53). Current findings in chronic schizophrenia patients are partly inconsistent, probably due to confounders such as different illness duration and (divergent) antipsychotic medications, in addition to a wide range of recently discovered microbiota correlates (98), all of which have an extensive impact on the compositional variation of human gut bacteria (99). These confounding factors can be avoided by studying microbiome alterations in unmedicated samples in the early stages of psychosis. A special emphasis should be placed on pre-clinical pathologies (such as high schizotypal traits), since clinical populations are medicated and often suffer from multi-morbidity. These findings will additionally contribute to answers around causality: if the gut microbiome is altered at very early stages, it will strengthen the evidence that changes to the gut microbiome commence prior to full disease pathology, rather than in response to disease. Longitudinal designs will help to track gut microbial patterns over time and if they are predictive of future memory impairments.

Quantitative microbiome profiling should be accompanied by multimodal neuroimaging of the hippocampus, peripheral markers of inflammation, stress, neuroplasticity, vagal afferent signaling, and thorough memory assessments. In accordance with the MAM model (20, 100), hippocampal perfusion, GABA and glutamate, as well as hippocampus activation during episodic memory processing represent measures of special interest. Machine learning and mediation analyses can be conducted to test relationships between the gut microbiota, hippocampus function and episodic memory performance. Causality (proof-of-principle) between gut microbiota, hippocampus measures and memory can be accomplished through FMT studies from patients to germ-free mice followed by *in vivo* microdialysis and behavioral/cognitive testing. The overall aim of this research line is to identify targets along the gut microbiota-brain axis for improved prognosis of memory impairments and the development of novel microbiota-targeted interventions to treat or prevent memory impairments in psychosis.

### Development of microbiota-targeted interventions

Identifying robust and reliable gut microbiota markers that mediate hippocampus-dependent memory performance is indispensable to test target engagement of potential novel interventions within early phase clinical studies (Figure 1). The previously established microbiota markers can be used to test target engagement of putative novel memory treatments such as for instance next-generation probiotics (NGP) (101) or endocannabinoids (e.g., CBD), which are interacting with the gut microbiome (102, 103) and have anti-(neuro)inflammatory and anti-(neuro)oxidative central properties (104). Target engagement studies can be conducted in healthy volunteers to validate proof of mechanism. Subsequent open-label studies in small clinical or general population samples will follow to test feasibility, safety and efficacy of compounds with high target engagement (proof-of-concept). This line of research is essential for designing more efficient biomarker-tailored drug trials, likely accelerating the development of new therapeutics for memory impairments.

The clinical efficacy of promising interventions must further be validated in large-scale randomized controlled trials (RCTs). The findings of these trials will also allow to disentangle relationships between intervention effects on gut microbiota- brain markers and outcome measures, providing predictive biomarkers for future patient stratification. Such stratification biomarkers will be established with state-of-the art machine learning algorithms to preselect those individuals who are most likely to respond to the interventions, e.g., by multiclass learning algorithms. Subsequent superiority trials can finally be conducted to compare the effects of biomarker-guided versus non-stratified trial designs.

## Conclusion

Elucidating gut microbiota–hippocampus synergisms constitute a paradigm shift in psychosis research and hold the promise of identifying novel targets for the development of memory interventions. This is of clinical relevance, as memory impairments are associated with a longitudinal risk of developing psychosis (2) and poor functional outcomes (3, 105) in high-risk individuals. In order to decipher the directionality and putative mechanisms of gut microbiota hippocampus interactions in psychosis we propose multimodal symptom driven studies.

A particular focus should be placed on people with the liability to develop psychosis prior to clinical manifestation, as this not only allows for larger and unbiased samples without typical confounders such as medication and illness duration, but also broadens the targeted symptoms and audience for early prevention strategies. By focusing future research on non-clinical subjects expressing subclinical symptoms such as schizotypal tendencies it will allow us to uncover the mechanisms of early psychosis development and to identify targeted points of intervention. There are currently no licensed interventions to prevent poor clinical outcomes in high-risk individuals (106–108) and whether antipsychotic drug treatment for prevention of psychosis in young individuals is justified remains controversial (109). As Zhang et al. (110) showed antipsychotics may even have negative effects on brain structure in CHR which could increase risk of psychosis onset. In combination with behavioral therapies such as cognitive remediation (111), microbial supplementation could offer accessible, pragmatical, and non-stigmatizing therapies for memory impairments in early stages of psychosis.

## Data availability statement

The original contributions presented in this study are included in the article/Supplementary material, further inquiries can be directed to the corresponding author.

## Ethics statement

Ethical review and approval was not required for the study on human participants in accordance with the local legislation and institutional requirements. Written informed consent for participation was not required for this study in accordance with the national legislation and the institutional requirements.

## Author contributions

AS: conceptualization of the theme. GI and SU: drafting the manuscript. GI, SU, and AS: critical revision of the manuscript for important intellectual content. All authors contributed to the article and approved the submitted version.
