# Supplementary material for: Elucidating gut microbiota–hippocampus interactions in emerging psychosis: A new perspective for the development of early interventions for memory impairments
Source: Front Psychiatry. 2023 Mar 23;14:1098019. doi: 10.3389/fpsyt.2023.1098019 (PMC10076719; doi:10.3389/fpsyt.2023.1098019)
Supplement: Supplementary file 1 [file Table_1.docx]

**Elucidating gut microbiota – hippocampus interactions in emerging psychosis: a new perspective for the development of early interventions for memory impairments**

Supplementary information

**Supplementary Table 1.** Hippocampus abnormalities along the progression of psychosis

|  |  | **Psychosis progression** | | | |
| --- | --- | --- | --- | --- | --- |
|  |  | Psychotic experiences / schizotypy (non-help seeking from the population) | CHR (help seeking in early detection services) | First-episode / early psychosis | Full psychosis |
| **Progression of hippocampus pathology** | Glutamate level | / | Increased (1) | Increased (2) | Increased (3) |
|  | Resting state perfusion | Increased (4) | Increased (5, 6) | Increased (7) | Increased (8, 9) |
|  | Activation during episodic memory | / | Reduced (10, 11, 12) | Reduced (13, 14) | Reduced (15, 16) |
|  | Grey matter volume | Reduced (17, 18) | Reduced (19) | Reduced (2, 20) | Reduced (21, 22) |

**References**

1. Provenzano FA, Guo J, Wall MM, Feng X, Sigmon HC, Brucato G, et al. Hippocampal Pathology in Clinical High-Risk Patients and the Onset of Schizophrenia. Biol Psychiatry. 2020;87(3):234-42.

2. Briend F, Nelson EA, Maximo O, Armstrong WP, Kraguljac NV, Lahti AC. Hippocampal glutamate and hippocampus subfield volumes in antipsychotic-naive first episode psychosis subjects and relationships to duration of untreated psychosis. Transl Psychiatry. 2020;10(1):137.

3. Kraguljac NV, White DM, Reid MA, Lahti AC. Increased hippocampal glutamate and volumetric deficits in unmedicated patients with schizophrenia. JAMA Psychiatry. 2013;70(12):1294-302.

4. Modinos G, Egerton A, McMullen K, McLaughlin A, Kumari V, Barker GJ, et al. Increased resting perfusion of the hippocampus in high positive schizotypy: A pseudocontinuous arterial spin labeling study. Hum Brain Mapp. 2018;39(10):4055-64.

5. Allen P, Chaddock CA, Egerton A, Howes OD, Bonoldi I, Zelaya F, et al. Resting Hyperperfusion of the Hippocampus, Midbrain, and Basal Ganglia in People at High Risk for Psychosis. Am J Psychiatry. 2016;173(4):392-9.

6. Allen P, Azis M, Modinos G, Bossong MG, Bonoldi I, Samson C, et al. Increased Resting Hippocampal and Basal Ganglia Perfusion in People at Ultra High Risk for Psychosis: Replication in a Second Cohort. Schizophr Bull. 2017.

7. McHugo M, Talati P, Armstrong K, Vandekar SN, Blackford JU, Woodward ND, et al. Hyperactivity and Reduced Activation of Anterior Hippocampus in Early Psychosis. Am J Psychiatry. 2019;176(12):1030-8.

8. Talati P, Rane S, Kose S, Blackford JU, Gore J, Donahue MJ, et al. Increased hippocampal CA1 cerebral blood volume in schizophrenia. Neuroimage Clin. 2014;5:359-64.

9. Schobel SA, Lewandowski NM, Corcoran CM, Moore H, Brown T, Malaspina D, et al. Differential targeting of the CA1 subfield of the hippocampal formation by schizophrenia and related psychotic disorders. Arch Gen Psychiatry. 2009;66(9):938-46.

10. Allen P, Seal ML, Valli I, Fusar-Poli P, Perlini C, Day F, et al. Altered prefrontal and hippocampal function during verbal encoding and recognition in people with prodromal symptoms of psychosis. Schizophr Bull. 2011;37(4):746-56.

11. Allen P, Luigjes J, Howes OD, Egerton A, Hirao K, Valli I, et al. Transition to psychosis associated with prefrontal and subcortical dysfunction in ultra high-risk individuals. Schizophr Bull. 2012;38(6):1268-76.

12. Valli I, Stone J, Mechelli A, Bhattacharyya S, Raffin M, Allen P, et al. Altered medial temporal activation related to local glutamate levels in subjects with prodromal signs of psychosis. Biol Psychiatry. 2011;69(1):97-9.

13. Achim AM, Bertrand MC, Sutton H, Montoya A, Czechowska Y, Malla AK, et al. Selective abnormal modulation of hippocampal activity during memory formation in first-episode psychosis. Arch Gen Psychiatry. 2007;64(9):999-1014.

14. Francis MM, Hummer TA, Vohs JL, Yung MG, Liffick E, Mehdiyoun NF, et al. Functional neuroanatomical correlates of episodic memory impairment in early phase psychosis. Brain Imaging Behav. 2016;10(1):1-11.

15. Achim AM, Lepage M. Episodic memory-related activation in schizophrenia: meta-analysis. Br J Psychiatry. 2005;187:500-9.

16. Ragland JD, Ranganath C, Harms MP, Barch DM, Gold JM, Layher E, et al. Functional and Neuroanatomic Specificity of Episodic Memory Dysfunction in Schizophrenia: A Functional Magnetic Resonance Imaging Study of the Relational and Item-Specific Encoding Task. JAMA Psychiatry. 2015;72(9):909-16.

17. Roalf DR, Quarmley M, Calkins ME, Satterthwaite TD, Ruparel K, Elliott MA, et al. Temporal Lobe Volume Decrements in Psychosis Spectrum Youths. Schizophr Bull. 2017;43(3):601-10.

18. Satterthwaite TD, Wolf DH, Calkins ME, Vandekar SN, Erus G, Ruparel K, et al. Structural Brain Abnormalities in Youth With Psychosis Spectrum Symptoms. JAMA Psychiatry. 2016;73(5):515-24.

19. Mechelli A, Riecher-Rössler A, Meisenzahl EM, Tognin S, Wood SJ, Borgwardt SJ, et al. Neuroanatomical abnormalities that predate the onset of psychosis: a multicenter study. Arch Gen Psychiatry. 2011;68(5):489-95.

20. Szeszko PR, Goldberg E, Gunduz-Bruce H, Ashtari M, Robinson D, Malhotra AK, et al. Smaller anterior hippocampal formation volume in antipsychotic-naive patients with first-episode schizophrenia. Am J Psychiatry. 2003;160(12):2190-7.

21. van Erp TG, Hibar DP, Rasmussen JM, Glahn DC, Pearlson GD, Andreassen OA, et al. Subcortical brain volume abnormalities in 2028 individuals with schizophrenia and 2540 healthy controls via the ENIGMA consortium. Mol Psychiatry. 2016;21(4):585.

22. Antoniades M, Schoeler T, Radua J, Valli I, Allen P, Kempton MJ, et al. Verbal learning and hippocampal dysfunction in schizophrenia: A meta-analysis. Neurosci Biobehav Rev. 2018;86:166-75.
